# Hypoxia and Non-alcoholic Fatty Liver Disease

**DOI:** 10.3389/fmed.2020.578001

**Published:** 2020-10-23

**Authors:** Stephania C. Isaza, Elvira del Pozo-Maroto, Lucía Domínguez-Alcón, Liliam Elbouayadi, Águeda González-Rodríguez, Carmelo García-Monzón

**Affiliations:** Research Unit, Hospital Universitario Santa Cristina, Instituto de Investigación Sanitaria Hospital Universitario de La Princesa, Centro de Investigación Biomédica en Red de Enfermedades Hepáticas y Digestivas (CIBERehd), Madrid, Spain

**Keywords:** obstructive sleep apnea, NAFLD, hypoxia, hypoxia-inducible factors (HIFs), hepatosteatosis

## Abstract

Non-alcoholic fatty liver disease (NAFLD) is currently the most common chronic liver disease worldwide and comprises varied grades of intrahepatic lipid accumulation, inflammation, ballooning, and fibrosis; the most severe cases result in cirrhosis and liver failure. There is extensive clinical and experimental evidence indicating that chronic intermittent hypoxia, featuring a respiratory disorder of growing prevalence worldwide termed obstructive sleep apnea, could contribute to the progression of NAFLD from simple steatosis, also termed non-alcoholic fatty liver or hepatosteatosis, to non-alcoholic steatohepatitis; however, the molecular mechanisms by which hypoxia might contribute to hepatosteatosis setup and progression still remain to be fully elucidated. In this review, we have prepared an overview about the link between hypoxia and lipid accumulation within the liver, focusing on the impact of hypoxia on the molecular mechanisms underlying hepatosteatosis onset.

## Introduction

Non-alcoholic fatty liver disease (NAFLD) is the most common chronic liver disease among adults and children around the world whose prevalence between diabetic and obese individuals is around 80% compared to 30–50% of the general population (1). While in most NAFLD patients this liver disease is usually asymptomatic, only presenting a simple accumulation of fat in the hepatocyte called hepatosteatosis, 44–59% can progress to a more advanced form of liver injury termed steatohepatitis (NASH), featured by inflammation and ballooning with varied stages of fibrosis, which in turn can lead to more severe conditions of liver disease such as cirrhosis, portal hypertension, and, ultimately, hepatocellular carcinoma (2).

Clinical and experimental evidences suggest that hypoxia may play an important role in the pathophysiology of NAFLD. In that regard, the obstructive sleep apnea syndrome (OSA), a common disorder affecting 1–4% of the general population and 25–35% of obese individuals, is characterized by recurrent apnea or hypopnea episodes during sleep, leading to nocturnal intermittent hypoxia (IH), and it has been associated with all the components of metabolic syndrome, including NAFLD (3–5). However, the molecular mechanisms by which hypoxia might contribute to NAFLD setup and progression still remain to be fully elucidated. In this review, we have prepared an overview about the link between hypoxia and lipid accumulation within the liver, focusing our attention on how hypoxia regulates hepatic lipid metabolism and how, through these metabolic effects, it could contribute to the onset of the early phase of NAFLD called hepatosteatosis.

## NAFLD Pathogenesis

The mechanisms underlying NAFLD onset and progression are complex and multifactorial. Different theories have been formulated, leading initially to the “two hits hypothesis” (6). The appearance of hepatosteatosis, defined as the presence of fat in more than 5% of hepatocytes, is considered the first “hit.” The second “hit” involves factors triggering inflammation, hepatocellular damage, and fibrosis, leading to NASH. However, nowadays, a multiple-hit hypothesis has been postulated for NAFLD pathogenesis, which recapitulates the complexity of the human NAFLD where multiple parallel factors are implicated in the development and progression of the disease (7). Considering the first “hit,” it is well known that hepatosteatosis results from an imbalance between hepatic free fatty acid (FFA) uptake, *de novo* lipogenesis, lipid oxidation, and lipid export *via* very low-density lipoprotein (VLDL) particles (8). This is a crucial phase in NAFLD outcome because an excessive content of FFAs and their metabolites triggers lipotoxicity in hepatocytes, leading to progression from hepatosteatosis to the more advanced forms of NAFLD, such as NASH (9). However, the key molecular pathways driving hepatosteatosis are not completely defined, but there is a growing scientific evidence indicating that hypoxia-inducible factors contribute to hepatosteatosis onset.

## Molecular and Cellular Consequences of Hypoxia on Hepatosteatosis Setup

Oxygen is so highly required in the cellular machinery that its lack implicates a quick response in order to adapt the cell to this new situation. This response is mediated by the hypoxia-inducible factors (HIFs), which are composed of two subunits—HIFα and HIFβ–considering HIF1α and HIF2α as the best characterized HIFα subunits (10). In the presence of normal oxygen levels, three distinct iron-dependent enzymes called “prolyl hydroxylase domain” (PHD) proteins are able to hydroxylate two specific prolyl residues in the HIFα subunit. This hydroxylation compromised the HIFα subunit's stability since it will be recognized and ubiquitinated by the von Hippel Lindau (VHL) protein for further proteasomal degradation. When the oxygen levels decrease, PHDs become inactive, so HIFα subunits are stabilized and translocated into the nucleus, where they interact with the constitutively expressed HIFβ subunit and other factors such as CBP/p300. This transcriptional complex interacts with the hypoxia response element (HRE) and leads to gene expression induced by HIFs, which is cell- and tissue-dependent (11).

Different experimental approaches have been used to determine the effects of hypoxia on NAFLD development. In this regard, it has been proposed that hypoxia signaling is involved in the regulation of hepatic lipid metabolism, given to both PHDs and HIFs a major role in this process. There is convincing experimental evidence that hypoxia induces lipid accumulation in mouse livers and human hepatocytes. Regarding the role of PHDs, whole-body *Phd1* gene inactivation promoted hepatosteatosis in mice fed a low-fat diet (12), and likewise the combined genetic deficiency of PHD2 and PHD3 led to severe hepatosteatosis (13), but, interestingly, the presence of a single *Phd1* or *Phd3* allele reduced liver fat content (14). Moreover, genetic deficiency of PHD2 protected mice against diet-induced hepatosteatosis (15), whereas oral administration of a pan-PHD inhibitor improved metabolic dysfunction, but was unable to reduce hepatosteatosis in wild-type mice fed a high-fat diet (HFD) (16), suggesting that the potential beneficial effects of pharmacologic PHD inhibition on hepatosteatosis should target only PHD2. In any case, the role of PHDs on hepatosteatosis onset still remains to be fully elucidated and deserves further investigation.

Downstream of PHDs which constitute the first oxygen sensors, HIFs are the key mediators of the cellular transcriptional response to hypoxia, regulating the expression of more than 300 genes involved in many biologic processes such as angiogenesis, erythrocytosis, and glucose and lipid metabolism, among others (17). Regarding the role of HIFs on hepatosteatosis setup, some experimental studies revealed that both HIF1α and HIF2α were involved in hypoxia-induced lipid accumulation in hepatocytes, whereas other studies showed HIF2α as the major regulator of hepatic lipid metabolism because the absence of HIF2α, but not HIF1α, protected against lipid accumulation in the livers from mice lacking the *Vhl* gene (18, 19). Supporting the latter, we have just demonstrated that HIF2α induced CD36 expression and function, the major driver of FFA uptake, triggering lipid accumulation in hepatocytes *in vitro* and *in vivo*, thus contributing to the hepatosteatosis onset (20). In this line, it has been demonstrated that the development of steatosis in hypoxic HepG2 cells is a consequence of increased HIF2α, which upregulated the hepatic expression of the adipose differentiation-related protein (ADRP), also involved in FFA uptake (21). It has also been reported that hypoxia-induced HIF2α stabilization led to β-oxidation suppression *via* PPARα in fat-laden hepatocytes (22). Moreover, oxygen therapy ameliorated hepatic steatosis induced by HFD in mice by reducing hepatic HIF2α and lipogenic gene expression (23). Taken together, the results derived from these studies suggest that HIF2α increases hepatic FFA uptake and *de novo* lipogenesis as well as decreases mitochondrial β-oxidation.

Regarding HIF1α, a number of experimental studies in distinct murine models have shown that either systemic or hepatic *Hifa* genetic deletion or HIF1α antisense oligonucleotide treatment decreased hepatosteatosis, suggesting the potential of HIF1α inhibition for the treatment of NAFLD (24, 25). Conversely, other studies have revealed that HIF1α protected against alcohol or choline deprivation-induced fatty liver (26, 27), so further investigations are needed to clarify the impact of HIF1α in hepatosteatosis setup.

## Experimental Evidences Linking Intermittent Hypoxia to NAFLD

Besides the existence of an epidemiological relationship between OSA and NAFLD, there is emerging evidence indicating that IH featuring OSA contributes to NAFLD onset and progression, but the underlying molecular mechanisms are not fully defined. One of the main purposes of the studies published concerning this issue was to determine the gene expression profile involved in lipid metabolism under IH conditions, considering HIFs as the main drivers in this regulation. As has been stated before, HIFs are the master regulators of the cellular response to hypoxic stress (10). In this regard, it has been experimentally demonstrated that IH could be a major trigger for NAFLD. Indeed, IH directly induced hepatosteatosis through the administration of repetitive brief periods of hypoxia and reoxygenation mimicking OSA in animal models (28). Several studies from the same research group demonstrated that IH promoted hepatic lipid accumulation mainly by inducing *de novo* lipogenesis. They firstly established that IH caused dyslipidemia and hepatosteatosis by activating the SREBP1c–SCD1 signaling pathway in the liver of lean mice (29). Later on, they demonstrated that partial *Hifa*-deficient mice were protected against hepatosteatosis and hyperinsulinemia induced by IH (30). In addition, they also showed that partial HIF1α knockdown modulated SREBP1c, SREBP cleavage-activating protein (SCAP), and SCD1 expressions in mice under IH, confirming the previous hypothesis (30, 31). Regarding HIF2α, a recent study revealed that IH exacerbated hepatosteatosis in mice fed HFD, which showed hepatic HIF2α overexpression along with a decreased β-oxidation and an enhanced *de novo* lipogenesis (22). Interestingly, silencing of *hif-2*α reduced lipid accumulation in hypoxic hepatocytes (20, 22), pointing out to HIF2α as a key driver in hepatosteatosis setup. Recently, we have observed an upregulated expression of CD36, together with an increased triglyceride content, in livers from mice exposed to IH, pointing out that IH may also modulate FFA uptake (32).

## Clinical Evidences Linking OSA to NAFLD

As stated above, OSA has been linked to lipid accumulation in the liver (33). In this regard, well-designed meta-analysis and systematic reviews have pointed out the relationship between OSA and NAFLD, stating that OSA is associated with an increased prevalence of hepatosteatosis, NASH, and fibrosis, independently of well-known risk factors such as age, sex, body mass index, or waist circumference (3, 34). Additional clinical studies and clinical trials have reinforced this notion, and their more relevant findings are summarized in Table 1. Notably, a study has demonstrated that OSA patients were three times more likely to have NASH compared with subjects without OSA (41). Moreover, clinical evidence suggests a direct relationship between OSA and NAFLD severity (42, 45). Interestingly, low O_2_ saturation has been proposed as an important NAFLD risk factor in OSA patients: the lower the O_2_ saturation, the higher the NAFLD severity (37, 46). Indeed, in a large study comprising 1,285 patients with suspected OSA aimed to assess the potential relationship between OSA and NAFLD, a significant positive correlation between the severity of hypoxemia and serum markers of liver injury was observed (40).

**Table 1:** Principal clinical studies examining the impact of OSA on NAFLD.

**Study**	**Sample size (patients/controls)**	**Study design**	**Primary endpoints**	**Main findings**
Minville et al. (35)	226 adult OSA patients/0 controls	Cross-sectional study	NAFL and NASH by non-invasive tools and OSA by polysomnography	Tc90% was significantly associated with NAFL, but not with NASH.
Sundaram et al. (36)	25 adolescent NAFLD patients (15 with OSA/10 without OSA)	Cross-sectional study	NAFLD by liver histology and OSA by polysomnography	OSA was significantly associated with NAFL, NAS score, and fibrosis stage.
Cakmak et al. (37)	118 adult OSA patients/19 without OSA	Cross-sectional study	NAFLD by ultrasonography and OSA by polysomnography	AHI and ODI were significantly higher in NAFLD than in controls.
Benotti et al. (38)	269 obese adults with OSA/93 obese adults without OSA	Cross-sectional study	NAFLD by liver histology and OSA by polysomnography	OSA severity was associated with NAFLD only in patients without metabolic syndrome.
Jullian-Desayes et al. (39)	103 adult OSA patients treated with effective CPAP vs. sham CPAP	Randomized controlled clinical trial	NAFLD by non-invasive tools and OSA by lung function parameters	NAFLD did not improve after 6–12 weeks of effective CPAP treatment.
Trzepizur et al. (40)	1,170 adult OSA patients/115 adults without OSA	Cross-sectional study	NAFLD by non-invasive tools and OSA by respiratory recordings	OSA severity correlated with hepatosteatosis, but not with fibrosis.
Asfari et al. (41)	1,490,150 hospitalized OSA patients/29,222,374 non-OSA hospitalized patients	USA database study	OSA and NASH diagnosis by ICD-9 code in clinical records	NASH diagnosis was 3-fold more frequent among OSA patients than in non-OSA patients.
Jin et al. (42)	2,272 adult OSA patients (2007–2017)	Meta-analysis and systematic review	NAFLD by liver histology and OSA by polysomnography	OSA positively correlated with hepatosteatosis, ballooning, and fibrosis.
Kim et al. (43)	351 adult OSA patients on CPAP therapy	Institutional prospective database study	NAFLD by transaminases and APRI index and OSA by polysomnography	OSA patients with good adherence to 3 months CPAP therapy improved transaminases and APRI index (liver fibrosis).
Sundaram et al. (44)	Nine adolescent OSA patients on CPAP therapy/23 adolescent untreated OSA patients	Observational longitudinal study	NAFLD by transaminases and OSA by polysomnography	Effective 3 months CPAP therapy improved ALT.
Schwenger et al. (45)	49 obese adults with NAFLD/12 obese adults with normal liver	Cross-sectional study	NAFLD by liver histology and OSA by polysomnography	AHI positively correlated with liver inflammation.

*OSA, obstructive sleep apnea; NAFLD, non-alcoholic fatty liver disease; NAFL, non-alcoholic fatty liver or hepatosteatosis; NASH, non-alcoholic steatohepatitis; NAS, NAFLD activity score; AHI, apnea/hypopnea index; ODI, oxygen desaturation index; Tc90%, percentage of sleep time with oxygen saturation <90%; CPAP, continuous positive airway pressure; APRI, aspartate aminotransferase-to-platelet ratio index*.

OSA is especially prevalent among obese individuals, but IH may differently affect the liver and adipose tissue in obese patients as it has been strongly associated with liver damage, whereas, apparently, it has no effect on adipocyte morphology or adipose tissue macrophage accumulation (47). Several studies examining cohorts of obese patients with sleep apnea have found that IH is closely associated with NAFLD diagnosed using non-invasive tools (35), but, even more important, with the histological features of NASH including lobular inflammation, hepatic ballooning, and hepatic fibrosis (28). Interestingly, a dose–response relationship has been observed between the severity of nocturnal hypoxia and liver injury in obese patients in the absence of metabolic syndrome (38).

OSA has been related to pediatric NAFLD as well: it affects 68% of obese and 44% of non-obese children with NAFLD. In fact, a correlation between the severity of hypoxia and the severity of pediatric NAFLD has been observed since liver tissue infiltration by leukocytes and activated macrophages as well as fibrosis and liver apoptosis are increased in these patients (36).

Taking this background into account, it is conceivable that continuous positive airway pressure (CPAP), which is the first-line therapy for OSA patients, could be useful by attenuating IH-related deleterious effects. In this regard, there are clinical evidence that arterial hypertension and elevated circulating catecholamine levels, commonly seen in OSA patients, improve after CPAP treatment (39). Regarding NAFLD, contradictory reports have been published. It has been reported that CPAP treatment appeared to have no significant effect on OSA-related liver injury as well as on lipid and glucose metabolism (48); conversely, CPAP treatment in adult and adolescent patients with OSA caused an improvement in serum aminotransferase activity as well as an apparent regression of hepatic fibrosis (43). Therefore, the potential beneficial effects of CPAP therapy on cardiovascular complications and metabolic disorders, such as insulin resistance and NAFLD, associated with OSA remain to be fully elucidated, and studies in large well-designed clinical trials assessing the impact of CPAP therapy on NAFLD in patients with OSA patients are clearly needed.

## Conclusions

**Figure 1:**
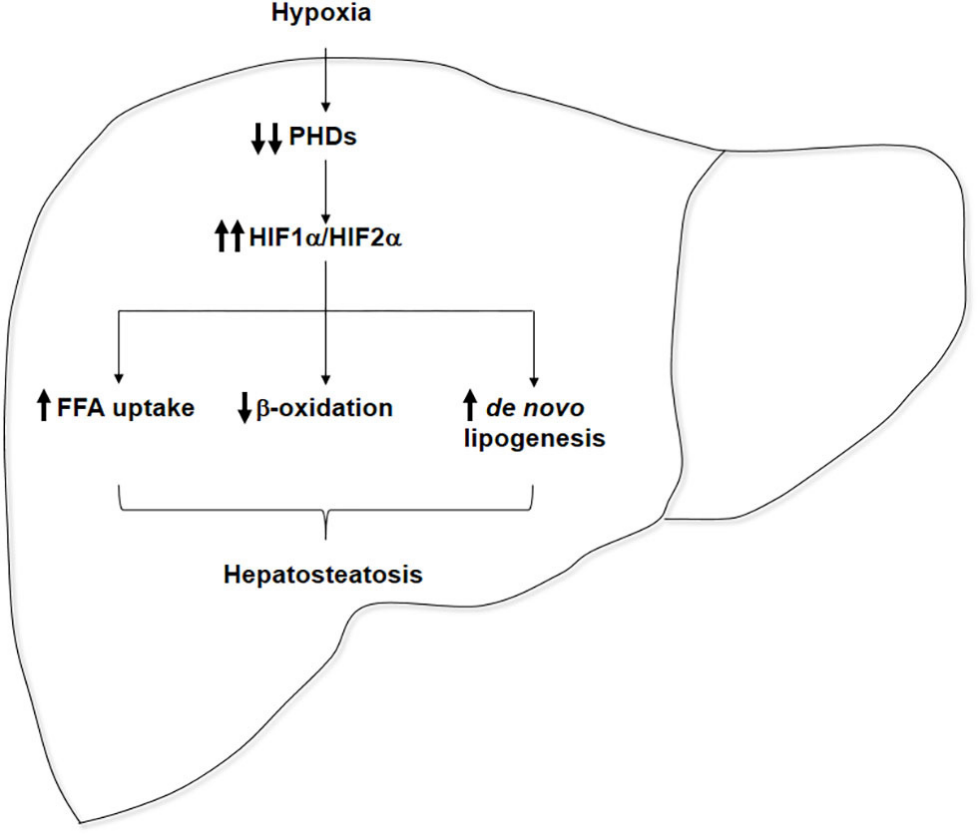
Pathophysiological role of hypoxia in hepatosteatosis onset. Hypoxia inactivates prolyl hydroxylase domains (PHDs), enhancing hepatic HIF1α and HIF2α expressions, which could contribute to hepatosteatosis onset by the upregulation of free fatty acid (FFA) uptake, the repression of FFA β-oxidation, and the stimulation of *de novo* lipogenesis.

Emerging evidence suggests that OSA may play a role in the onset of hepatic steatosis and in the progression of NAFLD. Several cross-sectional studies showed that the severity of IH in patients with OSA predicts the severity of NAFLD on liver biopsy. Different animal models have provided insights on the potential effects of hypoxia on the molecular mechanisms underlying NAFLD pathogenesis, which are graphically represented in Figure 1, showing that hypoxia upregulates both HIF1α and HIF2α in the liver, which may increase hepatic steatosis by the induction of *de novo* lipogenesis and FFA uptake and by the repression of FFA β-oxidation. However, the role of HIFs in the pathogenesis of IH-induced NAFLD is yet to be fully elucidated. Thus, multiple studies point out that IH featuring OSA may contribute to the progression of NAFLD, but definitive clinical studies and experiments in validated mouse models of NAFLD have yet to be done. Nevertheless, hypoxia could be considered as another “hit” among the “multiple parallel hits” that have been proposed as responsible for NAFLD setup and progression to NASH.

## Author Contributions

ÁG-R and CG-M organized review structure. SI, EdP-M, LD-A, LE, ÁG-R, and CG-M participated in the bibliographic search. SI, ÁG-R, and CG-M wrote the manuscript. All authors were involved in editing the paper and had final approval of the submitted and published versions.

## Conflict of Interest

The authors declare that the research was conducted in the absence of any commercial or financial relationships that could be construed as a potential conflict of interest.
